# Rewriting the blueprint of life by synthetic genomics and genome engineering

**DOI:** 10.1186/s13059-015-0689-y

**Published:** 2015-06-16

**Authors:** Narayana Annaluru, Sivaprakash Ramalingam, Srinivasan Chandrasegaran

**Affiliations:** Department of Environmental Health Sciences, Bloomberg School of Public Health, Johns Hopkins University, 615 North Wolfe Street, Baltimore, MD 21205 USA

## Abstract

Advances in DNA synthesis and assembly methods over the past decade have made it possible to construct genome-size fragments from oligonucleotides. Early work focused on synthesis of small viral genomes, followed by hierarchical synthesis of wild-type bacterial genomes and subsequently on transplantation of synthesized bacterial genomes into closely related recipient strains. More recently, a synthetic designer version of yeast *Saccharomyces cerevisiae* chromosome *III* has been generated, with numerous changes from the wild-type sequence without having an impact on cell fitness and phenotype, suggesting plasticity of the yeast genome. A project to generate the first synthetic yeast genome - the Sc2.0 Project - is currently underway.

## Introduction

Biology is now undergoing a rapid transition from the age of deciphering DNA sequence information of the genomes of biological species to the age of synthetic genomes. Scientists hope to gain a thorough mastery of and deeper insights into biological systems by rewriting the genome, the blueprint of life. This transition demands a whole new level of biological understanding, which we currently lack. This knowledge, however, could be obtained through synthetic genomics and genome engineering, albeit on a trial and error basis, by redesigning and building naturally occurring bacterial and eukaryotic genomes whose sequences are known.

Synthetic genomics arguably began with the report from Khorana’s laboratory in 1970 of the total synthesis of the first gene, encoding an artificial yeast alanine tRNA, from deoxyribonucleotides. Since then, rapid advances in DNA synthesis techniques, especially over the past decade, have made it possible to engineer biochemical pathways, assemble bacterial genomes and even to construct a synthetic organism [1–11]. Genome editing approaches for genome-wide scale alteration that are not based on total synthesis of the genome are also being pursued and have proved powerful; for example, in the production of a reduced-size genome version of *Escherichia coli* [4] and engineering of bacterial genomes to include many different changes simultaneously [8].

Progress has also been made in synthetic genomics for eukaryotes. Our group has embarked on the design and total synthesis of a novel eukaryotic genome structure - using the well-known model eukaryote *Saccharomyces cerevisiae* as the basis for a designer genome, known as ‘Sc2.0’. The availability of a fully synthetic genome will allow direct testing of evolutionary questions that are not otherwise approachable. Sc2.0 could also play an important practical role, since yeasts are the pre-eminent organisms for industrial fermentations, with a wide variety of practical uses, including production of therapeutic proteins, vaccines and small molecules through classical and well-developed industrial fermentation technologies.

**Fig. 1:**
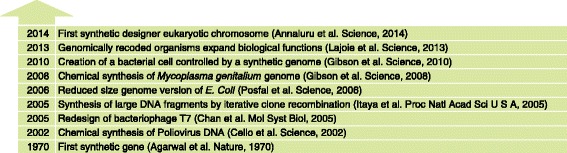
Timeline of publication milestones for synthetic genomics

This article reviews the current status of synthetic genomics, starting with a historical perspective that highlights the key milestones in the field (Fig. 1) and then continuing with a particular emphasis on the total synthesis of the first functional designer eukaryotic (yeast) chromosome, *synIII*, and the Sc2.0 Project. Genome engineering using nuclease-based genome editing tools such as zinc finger nucleases, transcription activator-like effector nucleases and RNA-guided CRISPR-Cas9 is not within the scope of this minireview (Box 1). Recent advances in gene synthesis and assembly methods that have accelerated the genome synthesis efforts are discussed elsewhere [12].

## Chemical synthesis of poliovirus cDNA (2002)

Viruses can be viewed as both chemical and ‘living’ entities. Since viral genomes are small, scientists wondered if it is possible to synthesize an infectious agent by in vitro chemical-biochemical means solely based on instructions from a known sequence. Poliovirus is an enterovirus of the Picornaviridae family and its sequenced genome comprises a single-stranded RNA genome of 7.5 kb in length. It replicates naturally in humans with high efficiency, occasionally causing the paralyzing and lethal poliomyelitis. The chemical synthesis of full-length Mahoney poliovirus cDNA *wt* PV1(M) by assembling oligonucleotides was first reported by Cello et al. [13]. The hierarchical strategy for synthesizing the genome of poliovirus involved three steps: (1) DNA fragments of 0.4-0.6 kbp length with overlapping complementary sequences at their termini were produced from purified oligonucleotides of approximately 70 nucleotides; (2) the 0.4-0.6 kbp fragments were then ligated into a plasmid vector to yield three larger DNA segments; (3) the assembly of a full-length cDNA carrying a phage T7 RNA polymerase promoter at the 5′ end was achieved from these three large overlapping DNA segments by cloning into a plasmid vector, using unique restriction sites. Several clones were sequenced to identify either the correct DNA segments or the segments containing small numbers of errors that could be eliminated, either by combining the error-free portions of segments by using an internal cleavage site or by standard site-directed mutagenesis. Nucleotide substitutions were engineered into the synthesized viral genome sPV1(M) cDNA as genetic markers to distinguish it from the wild-type sequence [13]. *De novo* synthesis of poliovirus from transcript RNA of sPV1(M) cDNA in a cell-free extract of uninfected HeLa cells indicated that the input synthetic RNA was translated and replicated in the cell-free extract and that newly synthesized RNA was encapsulated into newly synthesized coat proteins, resulting in infectious poliovirus [13]. This elegant work clearly established that it was possible to synthesize the genome of an infectious agent by in vitro chemical-biochemical means based on a known sequence.

## Refactoring bacteriophage T7 (2005)

Evolution by natural selection gives rise to complicated biological systems that are difficult to understand and manipulate. Wild-type T7, an obligate lytic phage that infects *E. coli*, is one such natural biological system. The T7 genome comprises a 39,937 bp linear double-stranded DNA molecule. It is an excellent model organism for discovering the primary genetic components of a natural biological system. The 57 genes coding for 60 proteins have been identified, of which only 35 have a known function. Of the 25 non-essential proteins, only 12 are conserved across the T7-like phage family. Driven by a desire to better understand how the different parts that comprise bacteriophage T7 work together to encode a functioning whole, scientists wanted to refactor the genome to a more structured design that is easy to manipulate and study.

Chan et al. [14] reported the redesign of bacteriophage T7 by improving its internal structure for future use, while simultaneously maintaining external system function; that is, physically separating the primary genetic elements that are essential for the functioning of the bacteriophage from the overlapping genetic elements that are non-essential for the viability of the phage. The T7.1 design goals were as follows. First, define a set of components that function during T7 development and for each element choose an exact DNA sequence to encode the element function. Second, avoid overlap between DNA sequences that encode different element functions. Third, assign only one function to the DNA sequence that encodes each element. Fourth, incorporate unique restriction sites for precise and independent manipulation of each element. Fifth, construct the T7.1 genome. Sixth, refactor the T7.1 genome to encode a viable bacteriophage. Each functional genetic element was defined, for example, as a promoter, protein-coding domain, ribosome binding site and so on. The authors replaced 11,515 bp of the 5′ part of the 39,937 bp wild-type bacteriophage T7 genome with 12,179 bp of engineered DNA using both synthetic DNA fragments and PCR-amplified T7 fragments, which contained all genetic elements of the 5′ end plus restriction enzyme sites. The resulting partially synthetic genome encoded a viable bacteriophage that appeared to maintain key features of the original while being simpler to model and making it easier to manipulate each genetic element encoding a function. This important work established that large regions of genomes encoding natural biological systems can be systematically redesigned and built anew.

## Synthesizing large DNA constructs by iterative clone recombination (2005)

While the smaller viral genomes, such as T7, are amenable to assembly by standard recombinant DNA techniques using synthetic or PCR-amplified precursor DNA fragments (see above), the assembly of larger bacterial genomes relies on recombination of the precursor DNA fragments in vivo in a host organism. For this approach to be successful, one has to be aware of the incompatibilities between the donor and the recipient host organism. Studies have shown that microbial genomes can be assembled in only evolutionarily divergent hosts (for example *Synechocystis* PCC6803 in *Bacillus subtilis*, or *Mycoplasma genitalium* in *S. cerevisiae*). In such instances the donor DNA remains transcriptionally silent without interfering with the viability of the host. The group of Itaya in Japan has used this approach to assemble a bacterial genome by serial integration of precursor DNA fragments directly into the *B. subtilis* genome. They cloned almost all of the 3.57 Mbp genome of the donor *Synechocystis* PCC6803 (a common and highly studied cyanobacterium) as a set of four separate fragments of approximately 800–900 kbp in a stepwise serial integration of PCR-generated precursor DNA fragments into the recipient *B. subtilis* genome [15]. This work showed that very large non-synthetic constructs could be produced from bacterial genomic DNA using in vivo methods. However, the resolution and activation of the synthetic donor genome is yet to be done.

Later, the Itaya group used the same approach to rebuild the full length mouse mitochondrial and rice chloroplast genomes from PCR-amplified precursors and recover the final synthetic DNA product as a circular episome [16]. Similarly, Holt et al. [17] achieved the reassembly of a fragmented donor genome of *Haemophilus influenzae* in a sequential manner into *E. coli*. This group used lambda Red recombination, which is an efficient system for *E. coli* chromosome engineering that uses electroporated linear DNA and a defective lambda phage to supply the functions needed for recombination. Using this technique, this group rebuilt two non-contiguous regions of *H. influenzae* genome totaling 190 kbp (approximately 10.4 % of the *H. influenzae* genome) as episomes in an *E. coli* host. However, both groups found that the bacterial recipient strains could not tolerate some sections of the donor genome, such as the rRNA operons and toxic genes.

## Chemical synthesis of *Mycoplasma genitalium* genome (2008)

The J Craig Venter Institute has pursued complete synthesis and assembly of a whole bacterial (*M. genitalium*) genome from chemically synthesized oligonucleotides. They reported successful synthesis and assembly of a 582,970 bp *M. genitalium* genome, a culmination of about 10 years of work [5]. In this case, the final complete donor *M. genitalium* genome was assembled in the recipient host *S. cerevisiae* (yeast). The synthetic genome was essentially the wild-type *M. genitalium* G37 sequence except for the disruption of the gene M408 with an antibiotic marker to block pathogenicity and allow for selection. A few watermarks were inserted at intergenic sites in order to identify the genome as synthetic. The hierarchical synthesis of the *M. genitalium* genome was done in three steps: (1) overlapping 5–7 kbp DNA fragments were assembled from chemically synthesized oligonucleotides; (2) the 5–7 kbp fragments were joined by in vitro recombination to yield intermediate 24 kbp, 72 kbp and 144 kbp fragments that were cloned into bacterial artificial chromosomes in *E. coli*; (3) the complete synthetic genome was assembled by homologous recombination in the yeast *S. cerevisiae*. Although a clone with the correct sequence was identified, Gibson et al. [5] did not demonstrate that the synthesized genome encodes a living bacterium. However, in subsequent work this was shown by the same group for a synthesized *Mycoplasma mycoides* genome (below) [10]. This impressive work established that chromosome-size DNA molecules could be constructed from chemically synthesized pieces.

## Synthesis and assembly of the *Mycoplasma mycoides* genome (2010)

Subsequently, Gibson et al. [10] reported the creation of a bacterial cell controlled by a chemically synthesized genome. A 1.08 Mbp *M. mycoides* genome was synthesized from known genome sequence; it was then transplanted into a closely related *Mycoplasma capricolum* recipient cell to form a new *M. mycoides* cell that was controlled solely by the synthetic genome. The chemically synthesized genome had several alterations compared with the wild-type CP001668, which included four watermark sequences, a designed 4 kbp gene deletion and nucleotide polymorphisms at 20 locations, 19 of which were from harmless mutations acquired during the assembly process. These 19 sequence alterations also served as polymorphic differences between the synthetic genome and the wild-type genome. The newly created cell had the expected phenotypic properties of *M. mycoides* and was capable of continuous self-replication [10].

The synthetic *M. mycoides* genome was assembled from 1,078 overlapping DNA cassettes in three steps: (1) DNA fragments of 1,080 bp, which were produced from overlapping synthetic oligonucleotides, were combined to form 109 larger DNA fragments of about 10 kbp; (2) these were then recombined in pools of 10 to create 11 DNA segments of about 100 kbp in length; (3) the 11 segments were recombined to form the complete *M. mycoides* genome. All assemblies were carried out by in vivo homologous recombination in yeast, except for two constructs that were enzymatically pieced together in vitro. The designed sequence was 1,077,947 bp in length.

The study also showed that a single base pair deletion in the essential gene *dnaA* could render the synthetic *M. mycoides* genome inactive, whereas large genome insertions and deletions in non-essential parts of the genome had no observable effect on viability. This foundational work provided a proof-of-principle experiment for producing cells based on computer-designed genome sequences, even though the synthetic genome had only very limited modifications from the naturally occurring *M. mycoides* genome.

## Minimal bacterial genome

The vast differences that exist in the genome sizes of bacterial species begs the question, ‘What is the minimal set of genes or the minimal genome [18] that is needed for cellular life?’ A corollary to this question is, ‘What is the minimal set of genes shared by all bacterial species through evolution?’ Using gene deletion methods, several groups have successfully produced smaller and increasingly stable, streamlined bacterial genomes. These studies, using what is known as the top-down approach, have shown that large proportions of bacterial genomes can be deleted without any major growth defects. Research on *E. coli* laboratory strain MDS42 has shown that almost 15.3 % of the genome could be eliminated without affecting its growth characteristics [4, 19, 20]. The deleted genes include the transposable elements and horizontally derived genes that have important roles under special environmental conditions. Further work has shown that as much as 22 % of the MDS42 genome could be eliminated without any major growth defects. Other groups have also reported successful genome reduction efforts in *Schizosaccharomyces pombe*, *B. subtilis* and *E. coli* [21–23].

*M. genitalium* is a bacterium with the smallest genome of any independently replicating cell; it encodes 485 protein coding genes of which 100 are non-essential when individually disrupted. The small size of mycoplasma genomes makes them a prime candidate for creating a minimal genome using the bottom-up approach of synthetic genomics [5]. The J Craig Venter Institute is working towards a minimal mycoplasma genome by exploring whether genes that can be disrupted individually without affecting the fitness could also be deleted globally. De novo genome synthesis offers the ability to simultaneously implement many directed changes to the natural genome by building and testing a variety of reduced genomes by genome transplantation in a closely related host strain. The bottom-up approach should make it possible to arrive at the minimal mycoplasma genome that enables cellular life.

## Expanding the genetic code of *E. coli* (2013)

**Fig. 2:**
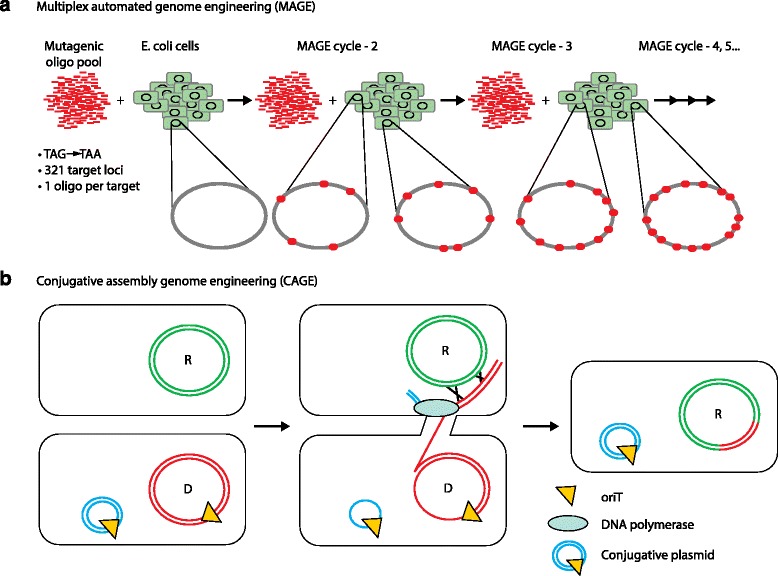
Multiplex automated genome engineering (MAGE) and conjugative assembly genome engineering (CAGE). **a** Use of MAGE (refer to [8] for more details) to replace all TAG codons with TAA in *E. coli*. **b** Use of CAGE (refer to [24] for more details) to incorporate a donor (D) into a recipient (R) genome. *oriT* in the donor genome serves as the transfer initiation point

Church, Isaacs and colleagues have used other genome-editing methods to alter the genetic code on a genome-wide scale in *E. coli*, thereby rewriting the genetic program. One approach, multiplex automated genome engineering (MAGE), allows for introduction of multiply targeted, small mutations through oligonucleotide-directed allelic replacement in an iterative manner (Fig. 2a; refer to [8] for more details). A second technique, conjugative assembly genome engineering (CAGE), allows for step-wise transfer of individually engineered genomic modules into a single genome (Fig. 2b; refer to [24] for more details). A combination of CAGE and MAGE was used to construct a recoded *E. coli* genome with an expanded genetic code [8, 24]. The translation-termination of the three stop codons (TAG, TAA and TGA) of the *E. coli* genetic code is mediated by two release factors, RF1 and RF2. RF1 recognizes the termination codons TAA and TAG, whereas RF2 recognizes TAA and TGA. The authors reasoned that replacing all TAG codons with synonymous TAA codons would abolish genetic dependence on RF1 and permit the newly reassigned TAA codons to be recognized by RF2. After removal of all genomic TAG codons, the *prfA* gene that codes for release factor 1 (RF1) was deleted. The authors hypothesized that this would enable them to test and leverage the redundancy of the genetic code and to provide a blank TAG codon that could be cleanly reassigned to a new function. The TAG codon was reintroduced along with an orthogonal set of aminoacyl-tRNA synthase and tRNA to encode a non-standard amino acid. The engineered *E. coli* incorporated non-standard amino acids into its proteins and showed enhanced resistance to bacteriophage T7 [25]. The Church group also recoded 13 codons in 42 highly expressed essential genes in *E. coli*, indicating that codon usage is quite flexible [26]. Recently, two laboratories have redesigned essential enzymes of *E. coli* with an altered genetic code by changing TAG codons to TAA. This confers metabolic dependence on non-standard amino acids for survival as a means for biocontainment of genetically modified organisms [27, 28].

## The first synthetic designer eukaryotic chromosome (2014)

The idea for designing and synthesizing a eukaryotic chromosome was initiated by our group in collaboration with Jef Boeke in 2005. The concept for hierarchically synthesizing a designer yeast chromosome was quite simple. First, design the synthetic chromosome incorporating all the desired changes based on the available wild-type chromosome sequence of *S. cerevisiae*. Second, compile the designed chromosome into pieces of about 10 kbp by including unique restriction sites at the 5′ and 3′ ends to enable further ligation of the 10 kbp pieces into segments of about 30–50 kbp. Synthesize these pieces of about 10 kbp using oligonucleotides from commercial vendors. Third, as yeast is highly recombinogenic, use an iterative strategy with alternating genetic markers to replace each 30–50 kbp segment of the wild-type sequence with the corresponding synthetic pieces, one at a time by homologous recombination in vivo in yeast.

The initial proof-of-principle experiment was performed in our laboratory by first designing and synthesizing a 30 kbp fragment of yeast chromosome *III* and then replacing the wild-type segment with the synthetic piece in yeast [29]. By 2007, the idea of synthesizing a eukaryotic chromosome had morphed into an ambitious project with the goal of rewriting wild-type *S. cerevisiae* Sc1.0 into a synthetic version, Sc2.0.

### Design principles for the synthetic yeast genome (Sc2.0)

Suggestions for the types of changes to be incorporated into Sc2.0 were obtained by Boeke from the community of yeast researchers. Only conservative changes were included, as more drastic changes might result in ‘dead’ yeast. The synthetic yeast should have the same fitness as the wild type and grow normally; this is an obvious minimal requirement for Sc2.0. The three design principles for the synthetic yeast genome are as follows: (1) it should result in a (near) wild-type phenotype and fitness; (2) it should lack destabilizing elements to avoid the synthetic yeast genome from being unstable or undergoing rearrangements; (3) it should have genetic flexibility to facilitate future studies [30].

How does one design a Sc2.0 genome that will facilitate future studies? Yeast contains about 6000 genes and almost 5000 of these are non-essential when disrupted individually [31]. As such, all the non-essential genes were flanked with loxPsym sites. Once a synthetic chromosome or the Sc2.0 genome is built, in theory, one could expose the synthetic yeast strains to Cre recombinase for various time intervals and look for survivors. PCR-Tag analysis (see *synIII* construction) and sequencing of the genomes of survivors would reveal what combinations of non-essential genes have been deleted from the starting Sc2.0 genome, leaving the survivors viable.

### *synIII* design

**Fig. 3:**
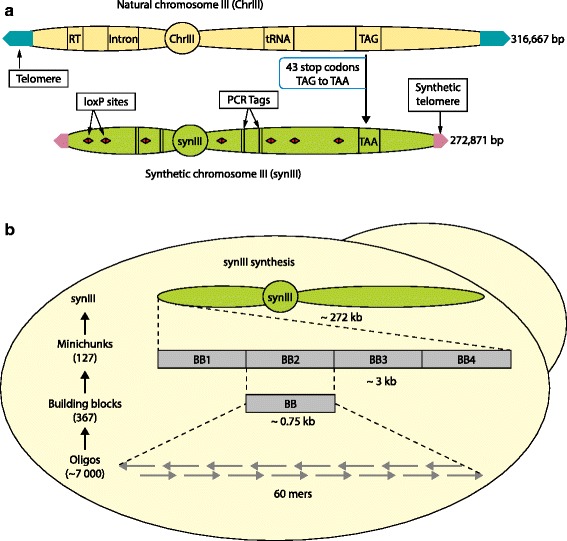
*synIII* design and synthesis. **a** *synIII* design. Twenty-one retrotransposons (*RT*) and seven introns were removed. Forty-three TAG stop codons were changed to TAA stop codons. Ninety-eight loxPsym sites were introduced to enable SCRaMbLE analysis. The two natural telomeres were replaced with shorter universal telomere caps. A single copy of essential tRNA gene *SUP61*, which codes for tRNASer (CGA), was deleted and moved to a tRNA neochromosome. Numerous PCR-Tags were incorporated into *synIII* to distinguish it from the natural counterpart. As a result, *synIII* is about 13.8 % smaller than the native yeast chromosome *III* (Box 2). For the complete set of additions, deletions and other genome modifications to *synIII*, see Annaluru *et al*. [32]. **b** *synIII* synthesis. *synIII* was constructed in three steps (shown in the flow diagram on the left, from bottom to top). In step 1, 750 bp building blocks (*BB*) were synthesized from 60-mer oligonucleotides at Johns Hopkins University by undergraduate students in the Build-A-Genome course [33]. In step 2, three to five BB were assembled into 2–4 kb minichunks by homologous recombination in *Saccharomyces cerevisiae* [35]. Adjacent minichunks were designed to encode overlap of one BB to facilitate downstream assembly. In step 3, direct replacement of native yeast chromosome *III* with pools of synthetic minichunks was performed. Eleven iterative one-step assemblies and replacements of native genomic segments of yeast chromosome *III* were carried out using pools of overlapping synthetic DNA minichunks, encoding alternating genetic markers (*LEU2* or *URA3*), which enabled complete replacement of native *III* with *synIII* in yeast [32]. The number of oligonucleotides, BBs, and minichunks needed to construct *synIII* are shown in parentheses. *SynIII* is 272,871 bp long, compared with the 316,667 bp long native yeast chromosome *III*

After a successful proof-of-principle experiment involving the design of a synthetic 30 kbp chromosome III fragment that was used to replace the native sequence in yeast, the sequence of the whole native chromosome *III* was edited in silico using Biostudio [32] to incorporate a series of deletions, insertions and base substitution changes to produce the desired ‘designer’ sequence (Box 2 and Fig. 3a). The synthetic version of chromosome *III* (known as *synIII*) also encodes a built-in recombination system called SCRaMbLE (synthetic chromosome rearrangement and modification by loxP-mediated evolution) to enable removal of the non-essential parts of the chromosome, and therefore streamline it, by inducing genomic alterations of the *synIII* strain using Cre recombinase [32]. As the result of these alterations, *synIII* (272,871 bp) is about 13.8 % smaller than the native chromosome *III* (316,667 bp) [32].

### *synIII* construction

**Fig. 4:**
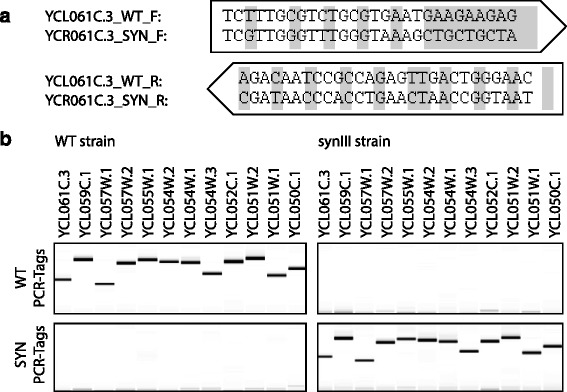
PCR-Tag analysis of a *synIII* segment. **a** The YCL061C.3 locus-specific PCR-Tag forward (F) and reverse (R) primers for the wild type (WT) and *synIII* are shown. The changes between the two are shaded. PCR-Tags are short pairs of recoded segments used as genetic markers to verify introduction of a synthetic sequence and removal of native sequence. Pairs of 25–28 bp sequences about 500 bp apart were recoded with synonymous codons such that >33 % of the bases were changed; the first and last base PCR-Tag primers were coded to be different between the WT and *synIII* sequences. **b** Agarose gel profiles of PCR-Tag analysis of a WT DNA segment and the corresponding synthetic *synIII* segment (YCL061C.3 to YCL050C.1). A virtual gel image was generated using LabChip GX software version 4.0.1418.0

The hierarchical workflow that was used to construct *synIII* (Fig. 3b) consisted of three major steps. In the first step, the 750 bp ‘building blocks’ (BBs) were produced starting from overlapping 60-mer to 79-mer oligonucleotides and assembled using standard PCR methods [33]. In a second step, the BBs were assembled into overlapping DNA ‘minichunks’ of approximately 2–4 kb using either the uracil-specific excision reaction [34] or cloning into a shuttle vector by homologous recombination in yeast *S. cerevisiae* [35–39]. In the USER approach, four to five BBs are used that each have a 5–13 bp sequence of the type A(N)_3_ T to A(N)_11_ T that overlaps with their adjoining neighbors and a vector. These BBs are amplified using forward and reverse primers containing a single uracil instead of the T and are then treated with USER enzymes (a mixture of uracil DNA glycosylase and the DNA glycosylase-lyase endonuclease VIII) to generate complementary single-stranded ends. The BBs are then ligated and cloned into *E. coli* to recover recombinants containing the assembled ‘minichunks’. The yeast homologous recombination cloning approach is much simpler, where four to five BBs each with 40 bp overlaps with their adjoining neighbors are assembled into a shuttle vector by direct transformation into the highly recombinogenic *S. cerevisiae*. This approach obviates the need for another round of PCR amplification of the BBs using primers containing uracil and the use of USER enzymes. Thus, as it turns out, all you need is yeast for minichunk assembly. In the third and final step, the adjacent minichunks for *synIII* were designed to overlap one another by one BB to facilitate further assembly in vivo by homologous recombination in yeast. Using an average of 12 minichunks and alternating selectable markers in each experiment, the native sequence of *S. cerevisiae III* was systematically replaced by its *synIII* counterpart in 11 successive rounds of transformation. PCR-Tag analysis (Fig. 4) and sequencing confirmed the identity of *synIII* [32]. The fact that the numerous design changes to the DNA sequence of the chromosome *III* had little or no impact on cell fitness and phenotype suggests the very pliable nature of the yeast genome [32].

### International consortium to synthesize the Sc2.0 genome 

A group of international scientists has taken up the synthesis of the Sc2.0 genome. The Beijing Genome Institute in China was the first to agree to synthesize four of the yeast chromosomes. Since then laboratories from various other countries have also joined the Sc2.0 effort to synthesize the remaining yeast chromosomes. Each participating laboratory is required to sign an Agreement with Johns Hopkins University (now with New York University). This arrangement leaves the control of the Sc2.0 project to Boeke, who is a yeast expert. Such a central organization is needed for the coordination of a huge undertaking such as Sc2.0 and for the distribution of yeast strains, reagents and experimental protocols. Participating laboratories have to raise their own funds from their own country to synthesize the allotted chromosome.

### What’s next for the yeast synthetic genome?

The *synIII* chromosome is about 2.5 % of the yeast genome and the changes that were made were all conservative, although numerous. These sequence alterations have not reduced the fitness of the yeast, which is encouraging in terms of the potential for future modifications. There are about 98 loxPsym sites in *synIII*, which scales to about 4000 loxPsym sites for the entire Sc2.0 genome. It is not yet clear how all of these loxPsym sites along with all the other modifications will ultimately affect the stability of the Sc2.0 genome and the viability of the synthetic yeast cell. The results from *synIII* are encouraging and the synthesis of a few more chromosomes will give us a better idea. Boeke’s laboratory is working on the assembly of the *synVI* chromosome using fragments of approximately 10 kbp from commercial vendors. Our laboratory is in the process of completing the assembly of the *synIX* chromosome. With the experience gained from the synthesis and assembly of *synIII*, we estimate that the construction of a chromosome about 1 Mbp could be done in 2–3 years.

**Fig. 5:**
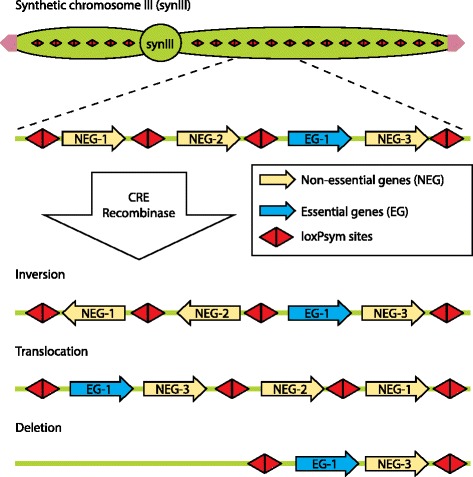
Synthetic chromosome rearrangement and modification by loxP-mediated evolution (SCRaMbLE) of the *synIII* strain. Examples of inversion, translocation and deletion products resulting from Cre recombinase treatment of *synIII* strain are shown

Once the Sc2.0 genome is built, an important focus will be to determine the minimal eukaryotic (yeast) genome. If two or more genes perform a similar function, can one be deleted? Which combinations of the 5000 yeast non-essential genes that are dispensable individually can be simultaneously removed? If we possess this knowledge, we will be able to achieve further reduction in the size of the Sc2.0 chromosomes and the genome. The plan is to use SCRaMbLE analysis to arrive at the minimal yeast genome (Fig. 5). This approach involves exposing Sc2.0 to Cre recombinase for various time intervals and looking for survivors. We reason that PCRTag analysis and sequencing the genomes of the survivors would reveal what combinations of non-essential genes have been deleted from the starting synthetic genome, leaving the survivors viable.

This pathway to the minimal yeast genome would represent a ‘top down’ approach since we start from the entire newly designed Sc2.0 genome and progressively delete increasing parts of the genome. However, to complicate matters, the essential and non-essential genes of the synthetic yeast are interspersed with one another. Because of this intertwining, SCRaMbLEing of the Sc2.0 is likely to result in dead yeast most of the time. Only yeasts with small deletions are likely to survive, making it difficult to deduce the minimal genome. Furthermore, due to the inherent symmetry of loxPsym sites, when two such sites are brought together by a Cre recombinase, it could result in an insertion, a deletion, an inversion or a translocation (Fig. 3). Moreover, there is also the possibility of interchromosomal rearrangements through the loxPsym sites, in addition to the expected intrachromosomal deletions, inversions, insertions and rearrangements. Analysis of such widely variant genomes from a population of survivors would involve time-consuming costly experimentation and complicated data analysis to decipher the minimal yeast genome. This hurdle could be overcome to some extent by performing SCRaMbLE analysis at the level of intermediate yeast strains, each possessing an individual synthetic chromosome. Thus, one could delineate a set of 16 minimal chromosomes for yeast. All of the reduced yeast chromosomes could then be combined into a final yeast strain to form a minimal eukaryotic genome.

## Conclusions and future perspectives

Recent literature reports make it clear that entire chromosomes and genomes can be designed, synthesized and incorporated into cells to produce synthetic organisms. However, to create a truly living, dividing synthetic cell from scratch by de novo synthesis, we need to know the minimal set of essential genes required for life and have a clear understanding of how each gene functions, and understand the regulatory mechanisms that are needed for harmonious gene function. It is very likely that the research on *Mycoplasma*, which have the smallest genomes among free-living cellular organisms, will be the first to lead to the delineation of the minimal set of genes required for life; this will be achieved either through stepwise iterative deletion of nonessential genes [40] or by de novo synthesis of several arbitrarily reduced genomes.

An international consortium of scientists is now working to synthesize the remaining 15 chromosomes of the yeast Sc2.0 genome, a model eukaryote. At this juncture, the best that one can say about Sc2.0 is that we are trying to rewrite Sc1.0 to Sc2.0, albeit with numerous conservative changes. The main stated purpose for designing and engineering of Sc2.0 is to improve our understanding of the evolution of eukaryotic genome structure and function.

Can the design rules be successfully applied across the entire yeast genome? Is the Sc2.0 genome worth doing? What are the potential industrial applications of Sc2.0? These are difficult questions to answer at this juncture. However, there is a critical need to develop alternative yeast strains as ‘chassis’ organisms for the production of pharmacologically important compounds such as artemisinin [41]. Will the ‘streamlined’ yeast strains resulting from in vitro evolution of the Sc2.0 genome be useful in this regard? Only time will tell. The total synthesis of a functional designer yeast chromosome represents an important milestone for eukaryotic synthetic biology. *synIII* work paves the way for other future synthetic genomics projects that seek to rewrite animal or plant chromosomes and genomes using specific design principles. The DNA synthesis technology and the genome engineering tools needed for such a major undertaking are currently available to scientists. However, it is too early to speculate about a minimal eukaryotic genome at this juncture.

In conclusion, recent breakthroughs in synthetic genomics have ushered in a new era for synthetic biology with the real potential to create a truly man-made living and dividing synthetic cell.

## Box 1. Genome engineering using programmable nucleases

Genome engineering by genome-editing tools depends on cellular responses to targeted chromosomal double-strand breaks (DSBs). Except for mouse cells, mammalian cells are recalcitrant to gene targeting [42]. Only one in a million treated cells undergoes homologous recombination (HR). However, it was discovered that stimulation of both local mutagenesis and incorporation of homologous donor sequences can be achieved by generating targeted DSBs, which was demonstrated most clearly with rare-cutting endonucleases [43]. The generation of a targeted DSB remained the rate-limiting step in the development of HR technology for mammalian cells until the creation of zinc finger nucleases (ZFNs) by our laboratory, which ushered in the breakthrough in programmable nucleases [44–47].

ZFNs: the first truly targetable reagents were the ZFNs, which showed that predetermined DNA sequences could be addressed for cleavage by protein engineering. ZFNs are formed by fusing a zinc finger protein (ZFP) that comprises a tandem array of ZF motifs [48] to the FokI non-specific cleavage domain [44,45]. Each ZF motif recognizes a DNA site of 3–4 bp [49]. Studies of the ZFN cleavage mechanism established that the preferred substrates are inverted repeats [50]. Soon afterwards, ZFN-induced DSBs were shown to stimulate HR in cells [51–53]. Because ZF motifs interact with and influence the recognition of their neighbors, the selection methods used to generate highly specific ZFPs for desired target sites are quite laborious and time-consuming. The commercial pricing of ZFNs was prohibitively expensive, putting it beyond the reach of small laboratories.

Transcription activator-like effector nucleases (TALENs): TALENs are based on the fusion of a different class of DNA-binding modules, called bacterial transcription activator-like effectors (TALEs), to the FokI cleavage domain [54]. Each TALE motif recognizes a single base and appears not to influence the sequence recognition of its neighbors [55,56]. Therefore, TALENs are relatively easier to engineer than ZFNs and they expanded the targeting capability of programmable nucleases. The fact that ZFNs and TALENs have been used to modify genomic sequences of more than 40 different organisms and cell types attests to the success of this approach to genome engineering. However, although they are cheaper than ZFNs, the commercial pricing of TALENs was still too expensive for smaller laboratories.

RNA-guided CRISPR-Cas9: the second technology platform for inducing a targeted DSB in cellular genomes is the RNA-guided nucleases (RGNs), which are based on the type II prokaryotic CRISPR-Cas9 system [57–61]. Unlike ZFNs and TALENs, which use ZF and TALE motifs, respectively, for DNA sequence recognition, the CRISPR-Cas9 system depends on RNA-DNA recognition, and its natural function is to combat invaders of bacteria and archaea, a testament to nature’s ability to solve problems several ways (compare restriction enzymes).The advantages of the CRISPR-Cas9 system are its ease of RNA design for new targets; the dependence on a single, constant Cas9 protein; and the ability to address many targets simultaneously with multiple guide RNAs. The CRISPR-Cas9 methodology is also very cheap and inexpensive, making it affordable for small laboratories. These have led to its wide adoption in research laboratories around the world.

These two technology platforms have equipped scientists with an unprecedented ability to modify cells and organisms almost at will, with wide-ranging implications across biology and medicine. However, both approaches have been shown to cut at off-target sites, with mutagenic consequences. Therefore, issues like efficacy, specificity and delivery are likely to drive selection of reagents for particular purposes. A word of caution about rushing to adopt CRISPR-Cas9 for human therapeutics and possibly for gene editing of human embryos: ease of design and use does not necessarily translate to safety. Therefore, human therapeutic applications of these technologies ultimately are likely to come down to risk versus benefit analysis and informed consent.

## Box 2. Modifications in *synIII* chromosome

- Elements removed: 10 transfer RNA genes, 21 Ty elements and/or derived long terminal repeats (LTRs), 7 introns, the silent mating loci *HML* and *HMR*, and subtelomeric sequences lying to the left of *YCL073C* and the right of *YCR098C* were removed [32]
- Elements relocated to extrachromosomal array: a single copy tRNA gene, *SUP61*, which codes for tRNASer (CGA) is essential to the yeast cell. Therefore, it was encoded in *trans* on a centromeric plasmid, which allowed deletion of the gene from *synIII* chromosome [32]
- Elements replaced: (1) TAG stop codons were replaced by TAA. Removal of the TAG stop codons from the synthetic genome will allow future genetic code manipulation [32]. (2) The telomeres were specified by a minimal ‘universal telomere cap’ comprising 305 bp of T(G)_1–3_ sequence. (3) Single synonymous codons were used to incorporate unique restriction sites (or delete sites) to facilitate *synIII* assembly. (4) Short stretches of synonymous codons (fewer than ten codons) were recoded to generate ‘PCR-Tags’ that serve as the basis for PCR primer design [30]. PCR-Tags are used to distinguish wild-type from synthetic sequence by selective PCR amplification
- Elements introduced: symmetrical loxP (loxPsym) sites were inserted in the 3′ UTR of all non-essential genes as well as at synthetic landmarks such as sites of LTR and tRNA deletion or flanking the centromere [32]. loxPsym sites lack the directionality of canonical loxP Cre recombinase sites and can align in two orientations. Therefore, both inversions and deletions are possible during SCRaMbLE using Cre recombinase [30]
- Elements not changed: gene order was preserved in *synIII* to prevent incorporation of a non-permissible configuration [32]. Induction of SCRaMbLE results in changes in gene order and chromosome structure [30]. All recovered SCRaMbLEd yeasts will have viable genome structures

## Abbreviations

BB: building block
bp: base pair
CAGE: conjugative assembly genome engineering
kb: kilobase
kbp: kilobase pair
MAGE: multiplex automated genome engineering
mpb: megabase pair
SCRaMbLE: synthetic chromosome rearrangement and modification by loxP-mediated evolution
TALEN: transcription activator-like effector nuclease
ZFN: zinc finger nuclease
