# Erratum to: Rewriting the blueprint of life by synthetic genomics and genome engineering

**DOI:** 10.1186/s13059-015-0703-4

**Published:** 2015-08-07

**Authors:** Narayana Annaluru, Sivaprakash Ramalingam, Srinivasan Chandrasegaran

**Affiliations:** Department of Environmental Health Sciences, Bloomberg School of Public Health, Johns Hopkins University, 615 North Wolfe Street, Baltimore, MD 21205 USA

## Erratum

In the version of this article that was originally published [1] there were errors in Figure three panel b (Figure 1b here) regarding the sizes of building blocks (BBs) and minichunks (wrongly denoted as 750 kb and 3000 kb, respectively). These mistakes, which were a result of clerical error that occurred during copy editing, have now been corrected online. The size of building block (BB) and minichunk is now correctly denoted as 0.75 kb and 3 kb, respectively.

**Fig. 1:**
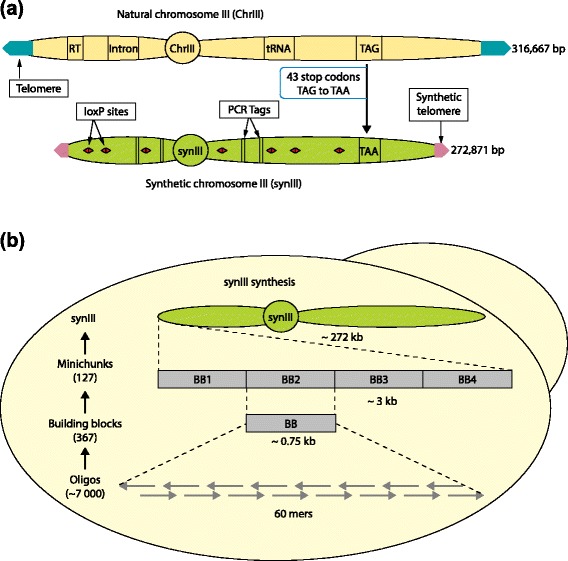
*synIII* design and synthesis. (**a**) *synIII* design. (**b**) *synIII* synthesis. This includes a revised version of Fig. 3(b) in the originally published article [1]. The size of the building block (BB) and minichunk is now correctly denoted as 0.75 kb and 3 kb, respectively

The journal apologizes for these errors.
